# Inflammatory Characteristics of Stenotic Aortic Valves: A Comparison between Rheumatic and Nonrheumatic Aortic Stenosis

**DOI:** 10.1155/2013/895215

**Published:** 2013-02-14

**Authors:** Lars Wallby, Thora Steffensen, Lena Jonasson, Mats Broqvist

**Affiliations:** ^1^Department of Clinical Physiology, Linköping Heart Centre, Linköping University Hospital, 581 85 Linköping, Sweden; ^2^Department of Pathology, University Hospital of Iceland, 101 Reykjavik, Iceland; ^3^Department of Cardiology, Linköping University Hospital, Linköping, Sweden

## Abstract

*Background*. Although our comprehension of nonrheumatic aortic stenosis (NRAS) has increased substantially during the last decade, less is known about the histopathology of rheumatic aortic stenosis (RAS). The aim of this study was to investigate rheumatic aortic stenosis by means of analyses previously used in nonrheumatic stenosis. *Material and Methods*. Valve specimens were obtained from 39 patients referred to hospital due to significant aortic stenosis. According to established macroscopic criteria the valves were divided into two groups consisting of 29 NRAS and 10 RAS valves. Mononuclear inflammatory cells and apolipoproteins were investigated using immunohistochemical analyses. *Results*. The localisation of calcification differed in tricuspid nonrheumatic valves when compared to bicuspid nonrheumatic and rheumatic valves. The RAS valves revealed a lower degree of T lymphocyte infiltration compared with the NRAS valves. Infiltration of macrophages was seen in all valves and there were no differences regarding deposition of apolipoprotein. *Conclusion*. Rheumatic and nonrheumatic aortic stenotic valves show a similar and significant chronic inflammation. The similarities regarding the localisation of calcification indicate that the valve anomaly/morphology can influence the pathogenesis of aortic stenosis. Finally, our findings highlight the question of a postinflammatory valvular disease of other causes than rheumatic fever.

## 1. Introduction

At the beginning of the 20th century, the incidence of rheumatic fever in the United States exceeded 100 per 100,000 population [1], and rheumatic heart disease was consequently the leading cause of heart valve illness. During the same century a gradual decrease in the incidence of rheumatic fewer was seen. The incidence ranged between 40 and 65 per 100,000 between 1935 and 1960 and is currently estimated at less than 2 per 100,000.

Improved socioeconomic conditions in the western world during the late half of the 20th century, with an increased life span, dramatically changed the aetiologic panorama of aortic stenosis. The so-called degenerative, nonrheumatic aortic stenosis (NRAS) has become the foremost cause of significant aortic valve obstruction. In adults undergoing aortic valve replacement for symptomatic aortic stenosis in the USA, nonrheumatic tricuspid aortic stenosis (NRAS-T) accounts for 51% of cases, bicuspid aortic stenosis (NRAS-B) for 36%, and rheumatic aortic stenosis (RAS) for 9% [2].

However, the presence of an aortic stenosis of rheumatic origin has been under debate. The sole pathognomonic feature of rheumatic valve disease, the Aschoff's granuloma, is virtually never found in heart valve tissue and thus, reliable diagnostic criteria is lacking. Presumed rheumatic stenotic aortic valves have been investigated in numerous studies with different approaches, all trying to master the nonattendant pathognomonic sign of rheumatic heart valve disease [3–6].

During the last two decades the knowledge of NRAS has increased considerably [7–12]. The aim of the present study was to compare RAS with NRAS, utilizing some of the analyses that we have previously performed on nonrheumatic heart valves [13, 14], and to investigate whether these analyses could contribute to our understanding of the stenotic disease of the aortic valve.

## 2. Material and Methods

Thirty-nine patients, consecutively accepted for surgery due to severe aortic valve stenosis, were enrolled in the study. Diagnosis was made by preoperative Doppler echocardiography. According to established macroscopic criteria for rheumatic heart valve disease [15, 16] the 39 stenotic aortic valves were divided into rheumatic aortic valves and nonrheumatic aortic valves.

### 2.1. Histopathological Analyses

After fixation in 10% formalin the valves were measured and examined macroscopically as described by Schoen [16]. A representative sample regarding calcification and fibrous thickening was taken from each valve. One section was taken from each cusp, each representing areas of thickening and calcification, but also including areas with minimal or no macroscopical changes, as calcification and fibrosis are not uniform processes. All grossly calcified valves were decalcified in 10% formic acid solution for 24 hours, then processed and cut in 4 *μ*m sections and stained with hematoxylin-eosin (H&E), van Gieson (VG), von Kossa, and Perls stains. Reference samples of all stains before and after decalcification were compared, thereby ensuring that the process of decalcification did not interfere with the staining and hence the result/interpretation of the staining.

Calcification was estimated by analysis of the gross specimen and by microscopic analysis of sections stained with the von Kossa stain. The extent of valvular calcification was arbitrarily graded as previously described by Subramanian et al. [17] as: 0 = absent, 1+ = mild, 2+ = moderate, or 3+ = severe. The degree of cusp thickening was arbitrarily graded as: 0 = absent, 1+ = increased valvular thickness only in the apposition area of the valve, 2+ = increased valvular thickness beyond the apposition area but not involving the entire valve, or 3+ = increased thickness of the valve by fibrosis in the entire valve or more limited areas of fibrosis which distorts the cusp shape. The degree of microcalcifications was semiquantitatively and arbitrarily categorized as: 0 = absent, trace = deposits not clearly visible on low power (25x), mild = scattered loose deposits or dense focal deposits covering <2 high-power fields (HPFs) (400x), moderate = dense deposits in >2 HPFs, and <6 HPFs, or severe = dense deposits in 6 or more HPFs. Furthermore, both the localisation of the calcification and the localisation of the fibrosis were investigated. The valves were evaluated for fresh haemorrhage on the H&E-stained sections and for old haemorrhage (haemosiderin deposits) with the Perls stain. Similar semiquantitative, arbitrary categorisation was used for both, which were graded as: 0 = absent, (+) = trace = haemosiderin deposits or fresh haemorrhage seen focally in 1 HPF (400x), mild = haemosiderin deposits or fresh haemorrhage seen in 2 HPFs, moderate = deposits seen in >2 HPFs, and <6 HPFs, severe = deposits in 6 or more HPFs. One reviewer, blinded both to the type of valve dysfunction and to the clinical history, performed all histological and immunohistochemical analyses.

### 2.2. Immunohistochemical Studies

For the determination of different mononuclear inflammatory cells, additional sections were stained with antibodies for CD3 (pan-T lymphocyte antigen, Dako, Carpinteria, CA, USA; 1:400), CD20 (pan-B lymphocyte antigen, Dako; 1:400), and CD68 (macrophage antigen, DakoCytomation, Glostrup, Denmark, 1:100). The valves were investigated regarding the presence and localisation of mononuclear cell infiltration. The degree of mononuclear cell infiltration was semiquantitatively and arbitrarily determined as previously described by Stratford et al. [18]: 0 = no inflammatory cells present, 1+ = occasional scattered cells or one group of 20 cells in a cusp section, 2+ = several groups of 20 cells or more in a cusp section, or 3+ = many groups of >20 cells or one group of 100 cells or more in a cusp section. 

For determination of the presence of apolipoprotein (apo) A-I and apo B, sections were stained with goat polyclonal antibody to human apo A-I (Abcam Ltd., UK; 1:800) and goat polyclonal antibody to human apo B (Abcam Ltd.; 1:800) and visualized with LSAB DakoCytomation K0690. Staining was by TechMate 500 according to standard protocol. The degree of apolipoprotein deposition was semiquantitatively categorized as follows: 0 = absent, 1+ = deposits in <5% of the cusp, 2+ = deposits in 5–25% of the cusp, 3+ = deposits in 26–50% of the cusp, 4+ = deposits in 51–75% of the cusp, 5+ = deposits in >75% of the cusp. If more than one cusp was involved, the one with the greatest deposits was scored.

### 2.3. Statistical Analyses

Comparisons between groups were performed using the *t*-test for parametric values and the Mann-Whitney test for nonparametric, nondependent samples. The data were considered significant at *P* < 0.05 level.

## 3. Results

Ten out of 39 valves (7 men, 3 women, mean age 64 ± 7 years) revealed postinflammatory changes with severely distorted and fused cusp margins (commissural fusion), resulting in a central triangular orifice. These valves were considered as RAS while the remaining 29 stenotic aortic valves were judged as NRAS. In 1/10 RAS, the valve was bicuspid, whereas the remaining 9/10 valves were tricuspid. Among NRAS valves, 12 valves (7 men and 5 women, mean age 67 ± 8 years) were considered to be bicuspid (NRAS-B) and 17 valves (7 men, 10 women, mean age 71 ± 7 years) to be tricuspid (NRAS-T). Clinical characteristics of RAS and NRAS groups are presented in Table 1.

Three out of 10 (30%) RAS patients and 2/29 (7%) NRAS patients had a history of rheumatic fever. Two RAS patients with a history of rheumatic fever also revealed a significant mitral valve stenosis, while the third RAS patient had been operated on 12 years earlier because of mitral valve stenosis. The remaining 7 RAS patients presented normal mitral valve leaflets with mostly mild mitral regurgitation.

### 3.1. Histopathological and Immunohistochemical Analyses

**Figure 1:**
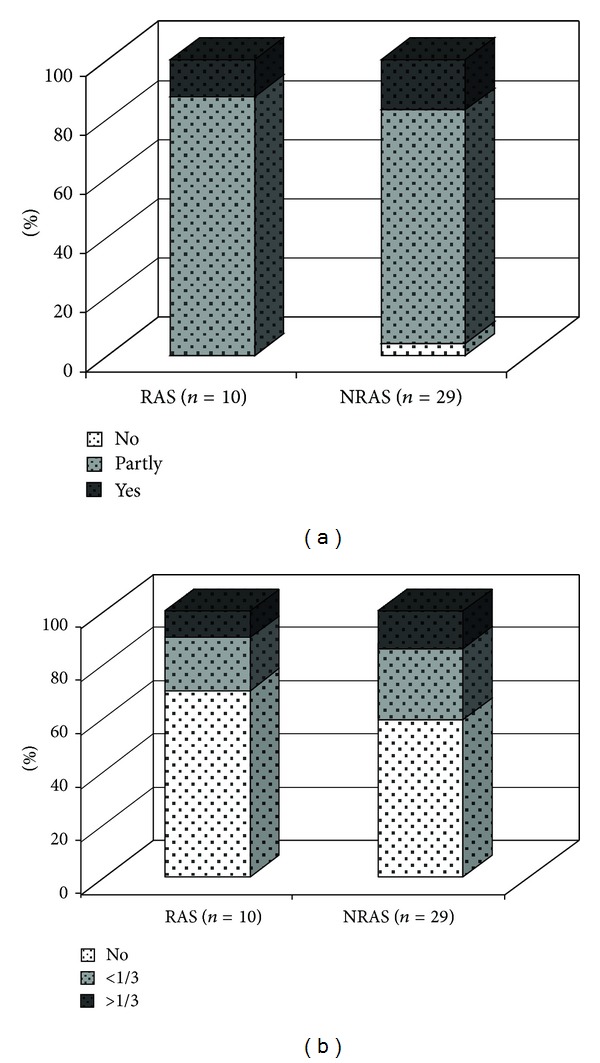
(a) Destruction of architecture in RAS as compared to NRAS; data given as percent (%) of (*n*). (b) Degree of neovascularisation in RAS as compared to NRAS, <1/3 = in the basal third of valve cusp, >1/3 = beyond basal third of valve cusp; data given as percent (%) of (*n*). RAS = rheumatic aortic stenosis, NRAS = nonrheumatic aortic stenosis.

Complete fibrous destruction of the normal layered architecture was seen in 3/10 (30%) RAS valves and in 5/29 (17%) NRAS valves (Figure 1(a)). However, several valves revealed partial destruction and only one valve, a nonrheumatic tricuspid valve, had completely normal architecture.

All the aortic valves, rheumatic as well as nonrheumatic, revealed mild to moderate cusp thickening. The fibrosis was in most cases localised diffusely. Only two valves, one rheumatic valve and one nonrheumatic, bicuspid valve, showed fibrosis at the apposition area. Three out of 10 (30%) RAS valves and 12/29 (41%) NRAS valves showed neovascularisation characterised by small irregular, thick walled and thin walled vessels within the basal third of the cusp (Figure 1(b)). Among valves revealing neovascularisation, neovessels were seen beyond the basal third of the valve in 1/3 RAS valves and 4/12 NRAS valves.

**Figure 2:**
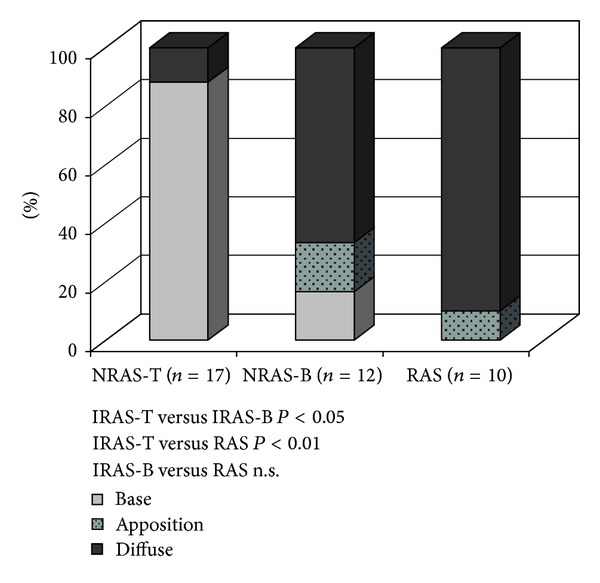
Localisation of calcification; number of valves given as percent (%) of (*n*). RAS = rheumatic aortic stenosis, NRAS = non rheumatic aortic stenosis.

Calcification was seen in all valves, ranging from mild to severe and without significant differences between the groups. Whereas the NRAS-T valves revealed calcification predominantly at the base of the cusp, the calcification in the majority of the RAS and NRAS-B valves was localised diffusely (Figure 2).

Due to our method of decalcification in 10% formic acid solution for 24 hours, we were unable to evaluate the von Kossa stain for mineralization, except in a few valves. 

**Figure 3:**
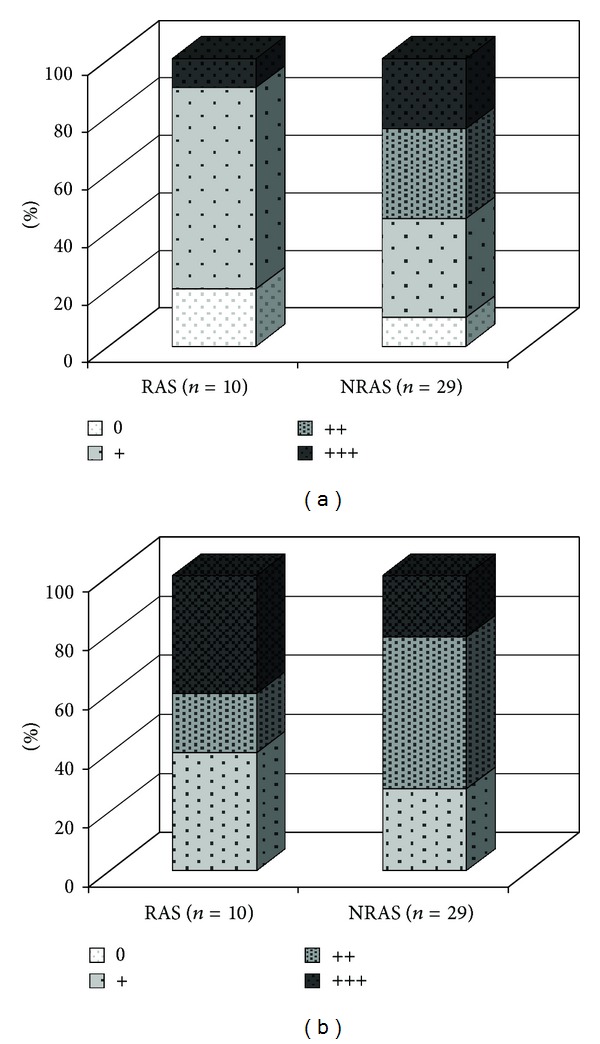
(a) Degree of lymphocyte infiltration in RAS as compared to NRAS. Number of valves given as percent (%) of (*n*). (b) Degree of macrophage infiltration in RAS as compared to NRAS. Number of valves given as percent (%) of (*n*). RAS = rheumatic aortic stenosis, NRAS = nonrheumatic aortic stenosis.

T lymphocytes were seen in 8/10 (80%) of the RAS and 26/29 (90%) of the NRAS valves. The degree of T lymphocyte infiltration was significantly lower in RAS than in NRAS valves (Figure 3(a)) while macrophages were abundantly present in both types of valves without any significant difference (Figure 3(b)). 

B lymphocytes were detected in 5/10 (50%) RAS valves, 6/12 (50%) NRAS-B valves, and 8/17 (47%) NRAS-T valves. Plasma cells were revealed in 5/10 (50%) RAS, 5/12 (42%) NRAS-B, and 3/17 (18%) NRAS-T valves.

**Figure 4:**
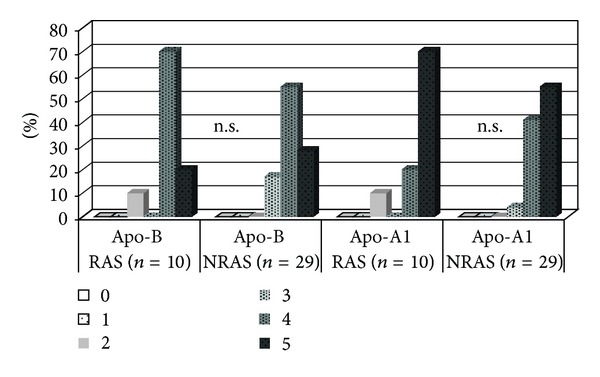
Degree of apolipoprotein B and A-1 deposition in RAS as compared to NRAS valves, data given as number of valves in percent (%) of (*n*). RAS = rheumatic aortic stenosis, NRAS = nonrheumatic stenosis.

Apolipoproteins were detected in all valves without any significant differences regarding the degree of either apo B or apo A-I (Figure 4).

## 4. Discussion

In the present study 10 stenotic aortic valves, defined as RAS on the basis of macroscopic postinflammatory fusion of the commissures, were investigated by means of histopathological and immunohistochemical techniques previously used for NRAS valves. According to our data the RAS valves revealed signs of chronic inflammation, including inflammatory cell infiltration, calcification, and deposition of apolipoproteins, in much the same manner as has previously been shown in NRAS valves. Minor differences were seen however.

Rheumatic heart valve disease is a well known, late, inflammatory and nonsuppurative complication of group A, streptococcal pharyngitis. In the western world rheumatic heart valve disease is seldom seen in its acute form, although the late, deforming, and chronic form still occurs sporadically in the population, especially in those with origins in countries with lower socioeconomic conditions.

The ratio of rheumatic (26%) versus nonrheumatic aortic valves in our patient cohort was greater than previously reported by Dare et al. (9%) [2], lower than reported by Waller et al. (43%) [19] and in the same range as reported by Passik et al. (24%) [20]. Regional and population-based variability in the incidence of rheumatic fever may account for some of the differences, but probably also various definitions of the rheumatic valve disease.

According to the literature, macroscopic features of RAS are thickened and fused cusps dominated by fibrosis. Calcification is very common according to Olsen [21] whereas Schoen and Sutton [22] describes mineralization as only a minor feature. The histological features are non-specific consisting of thickening due to collagen tissue, destroyed architecture, infiltration of inflammatory cells, and foci of calcification and sometimes ossification. The Aschoff granuloma, which occasionally can be found in the form of Aschoff and Anitschkow cells in valve tissue during the acute phase of rheumatic fever, is not described in chronic disease.

However, studies are lacking that investigate chronic rheumatic heart valve disease applying the inflammatory criteria used for acquired NRAS. Chopra et al. [23–25] described inflammatory changes in heart valve tissues from patients with rheumatic heart disease, from India and New Mexico, USA, but their studies mostly involved mitral valves and only a few aortic valves.

One reason why studies dealing with histology of RAS are sparse may be difficulties in how to define RAS contra NRAS. Goffin et al. [4] chose 63 patients with carefully verified history of rheumatic fever and used fibrotic scare tissue and neovascularisation as histopathological markers for diagnosis of rheumatic valve disease. One third of aortic valves showed definite rheumatic heart valve disease fulfilling both histopathological criteria, one third revealed only functional changes, and the remaining third showed only one of two criteria and was thus difficult to interpret. Even in cases of concomitant rheumatic mitral valve disease, the ratios were similar and Goffin concluded that rheumatic heart valve disease is principally a disease of the mitral valve. 

In our study, 10 patients were considered to have aortic valve disease of rheumatic origin, based on gross valvular pathology of thickened and fused cusps. Three of them revealed a history of rheumatic fever and, in addition, these individuals had a rheumatic disease of the mitral valve. On the other hand, the history of the remaining 7 subjects did not include anything that threw suspicion on a previous, serious streptococcal infection, neither did these subjects reveal any mitral valve disease. The fact that 7/10 RAS valves lacked a history of rheumatic fever and were devoid of echocardiographic signs of mitral valve stenosis highlights the question of a postinflammatory valvular disease of other cause than rheumatic fever [3, 26, 27].

Our findings are also in line with a previous study by Gallo et al. [3] who started out with 55 valves with postinflammatory scarring and divided them into three groups, patients with both streptococcal infection and rheumatic fever, patients with streptococcal infection without noncardiac major manifestations of rheumatic fever, and patients without any of these features. The pathological examination in that study was however unable to differentiate between the three groups, since all valves showed the same general pathological features. Gallo thus summarised that a postinflammatory valvular scarring of nonrheumatic aetiology must exist. 

Neovascularisation was seen in 12/29 (41%) NRAS and 3/10 (30%) RAS valves. Neovascularisation is considered a non-specific postinflammatory sign and can be seen in rheumatic heart valve disease, post endocarditis, atherosclerosis, diabetes, cancer, and extracardiac inflammatory conditions. Angiogenesis has also been demonstrated to be distinctly associated with the inflammatory process in NRAS [28]. Interestingly, as shown by Soini et al. [28], patients receiving statin therapy had significantly lower presence of neovessels.

Calcification constitutes a major feature of NRAS and is also found, though to a lesser extent, in RAS [16]. In our material we found moderate to severe calcification in the majority of RAS and NRAS valves. However, a distinct difference was seen regarding the localisation of calcification. Whereas NRAS-T mostly revealed calcification localised at the base of the cusps, NRAS-B valves as well as RAS valves revealed a significant higher proportion of diffuse calcification. The difference in calcification between NRAS-B and NRAS-T is previously described by Isner et al. [29]. They investigated 30 heavily calcified aortic valves and found nodular calcific deposits in 11/16 NRAS-T and diffuse calcification in 14/14 NRAS-B. One possible explanation to the similar localisation of calcification in RAS valves and NRAS-B valves could be that the fusion between two cusps, independent of underlying cause, give rise to similar mechanical and shear stress. 

The thesis of cuspal inequality as an underlying cause of valvular aortic stenosis has previously been presented by Roberts [6] and was not contradicted by a study by Vollebergh and Becker [30] showing that inequality in the tricuspid aortic valve is a rule more than an exception. A more or less pronounced inequality constituted by an unequal tricuspid valve, a congenital bicuspid valve, or an acquired fusion of cusps may thus lead to the development of chronic inflammation and valve stenosis. 

In general, the RAS valves revealed a somewhat lower degree of T-lymphocyte infiltration when compared to NRAS. Plasma cells were more commonly found in RAS when compared to NRAS, with the lowest share in NRAS-T valves. These figures suggest differences in the local inflammatory response although data are too limited to draw any conclusions.

Macrophages were equally abundant in RAS and NRAS valves, nor were there any significant differences regarding deposition of apolipoproteins. 

To summarize, most indices point towards a similar and significant chronic inflammation in both types of aortic stenotic valves, including neovascularisation, fibrosis, destruction of valve architecture, infiltration of macrophages, and deposition of apolipoproteins. The similarities regarding the localisation of calcification in RAS and NRAS valves also indicate that the valve anomaly/morphology, via its mechanical and shear stress properties, can influence the pathogenesis of aortic stenosis. Finally, our findings raise the hypothesis that other inflammatory conditions rather than rheumatic fever may give rise to the fusion of aortic cusps.

The condition involving calcification, infiltration by inflammatory cells, and deposition of lipoproteins resembles the inflammatory process of atherosclerotic disease [7–9, 11, 12, 31, 32]. In atherosclerosis, a cholesterol-lowering and anti-inflammatory regimen is considered a cornerstone of treatment. The similarities between calcific aortic valve disease and atherosclerosis, both from a histopathological and epidemiological point of view, raise the question whether it is also possible to prevent or slow disease progression of calcific aortic valve disease by cholesterol-lowering and anti-inflammatory therapy. Further and ongoing studies on this issue might give an answer to this question.

### 4.1. Study Limitations

The present study is a small descriptive and comparative study of RAS and NRAS. It focuses on possible differences in histopathology, including inflammatory features, but does not demonstrate any mechanistic information regarding the pathogenesis of RAS and NRAS.

## Figures and Tables

**Table 1:** Clinical characteristics in RAS and NRAS groups.

Characteristic	RAS (*n* = 10)		NRAS (*n* = 29)
Mean age ± SD (years)	64 ± 7	n.s.	69 ± 7
Male gender, *n* (%)	7 (70)	n.s.	14 (48)
Current/ex-smokers	1/7	<0,05	3/6
Hypertension, *n* (%)	1 (10)	n.s.	5 (17)
Diabetes, *n* (%)	0	n.s.	3 (10)
Angina pectoris, *n* (%)	5 (50)	n.s.	19 (66)
Prior myocardial infarction, *n* (%)	1 (10)	n.s.	2 (7)
Prior cerebrovascular event, *n* (%)	1 (10)	n.s.	2 (7)
Peripheral arterial disease, *n* (%)	1 (10)	n.s.	1 (3)
Coronary angiography, *n* (%)	9 (90)	n.s.	29 (100)
Angiographically verified coronary artery disease, *n* (%)	5 (50)	n.s.	9 (31)

## References

[1] Dajani AS, Zipes DP, Bonow RO, Mann DL (2005). Rheumatic fever. *Braunwald's Heart Disease*.

[2] Dare AJ, Veinot JP, Edwards WD, Tazelaar HD, Schaff HV (1993). New observations on the etiology of aortic valve disease: a surgical pathologic study of 236 cases from 1990. *Human Pathology*.

[3] Gallo P, Tonelli E, Marino B (1990). Postinflammatory scarring of cardiac valves of rheumatic and nonrheumatic etiology. *The American Journal of Cardiovascular Pathology*.

[4] Goffin YA, Leclerc JL, Primo GC (1984). Histopathology of the aortic valve in patients with a previous history of acute rheumatic fever. An analysis of 63 surgical specimens. *Acta Cardiologica*.

[5] Roberts WC (1970). Anatomically isolated aortic valvular disease. The case against its being of rheumatic etiology. *The The American Journal of Medicine*.

[6] Roberts WC (1970). The structure of the aortic valve in clinically isolated aortic stenosis: an autopsy study of 162 patients over 15 years of age. *Circulation*.

[7] O’Brien KD, Kuusisto J, Reichenbach DD (1995). Osteopontin is expressed in human aortic valvular lesions. *Circulation*.

[8] O’Brien KD, Reichenbach DD, Marcovina SM, Kuusisto J, Alpers CE, Otto CM (1996). Apolipoproteins B, (a), and E accumulate in the morphologically early lesion of “degenerative” valvular aortic stenosis. *Arteriosclerosis, Thrombosis, and Vascular Biology*.

[9] Olsson M, Dalsgaard CJ, Haegerstrand A, Rosenqvist M, Ryden L, Nilsson J (1994). Accumulation of T lymphocytes and expression of interleukin-2 receptors in nonrheumatic stenotic aortic valves. *Journal of the American College of Cardiology*.

[10] Olsson M, Rosenqvist M, Nilsson J (1994). Expression of HLA-DR antigen and smooth muscle cell differentiation markers by valvular fibroblasts in degenerative aortic stenosis. *Journal of the American College of Cardiology*.

[11] Olsson M, Thyberg J, Nilsson J (1999). Presence of oxidized low density lipoprotein in nonrheumatic stenotic aortic valves. *Arteriosclerosis, Thrombosis, and Vascular Biology*.

[12] Otto CM, Kuusisto J, Reichenbach DD, Gown AM, O’Brien KD (1994). Characterization of the early lesion of “degenerative” valvular aortic stenosis: histological and immunohistochemical studies. *Circulation*.

[13] Wallby L, Janerot-Sjöberg B, Steffensen T, Broqvist M (2002). T lymphocyte infiltration in non-rheumatic aortic stenosis: a comparative descriptive study between tricuspid and bicuspid aortic valves. *Heart*.

[14] Lars W, Thora S, Mats B (2007). Role of inflammation in nonrheumatic, regurgitant heart valve disease. A comparative, descriptive study regarding apolipoproteins and inflammatory cells in nonrheumatic heart valve disease. *Cardiovascular Pathology*.

[15] Pomerance A (1972). Pathogenesis of aortic stenosis and its relation to age. *The British Heart Journal*.

[16] Schoen FJ (1987). Surgical pathology of removed natural and prosthetic heart valves. *Human Pathology*.

[17] Subramanian R, Olson LJ, Edwards WD (1984). Surgical pathology of pure aortic stenosis: a study of 374 cases. *Mayo Clinic Proceedings*.

[18] Stratford N, Britten K, Gallagher P (1986). Inflammatory infiltrates in human coronary atherosclerosis. *Atherosclerosis*.

[19] Waller B, Howard J, Fess S (1994). Pathology of aortic valve stenosis and pure aortic regurgitation. A clinical morphologic assessment—part I. *Clinical Cardiology*.

[20] Passik CS, Ackermann DM, Pluth JR, Edwards WD (1987). Temporal changes in the causes of aortic stenosis: a surgical pathologic study of 646 cases. *Mayo Clinic Proceedings*.

[21] Olsen EGJ (1980). *The Pathology of the Heart*.

[22] Schoen F, Sutton M, Virmani R, Atkinson J, Fenoglio J (1991). Contemporary pathologic considerations in valvular heart disease. *Cardiovascular Pathology*.

[23] Chopra P, Bhatia ML (1992). Chronic rheumatic heart disease in India: a reappraisal of pathologic changes. *The Journal of Heart Valve Disease*.

[24] Chopra P, Tandon HD, Raizada V, Gopinath N, Butler C, Williams RC (1983). Comparative studies of mitral valves in rheumatic heart disease. *Archives of Internal Medicine*.

[25] Raizada V, Williams RC, Chopra P (1983). Tissue distribution of lymphocytes in rheumatic heart valves as defined by monoclonal anti-T cell antibodies. *The American Journal of Medicine*.

[26] Baandrup U (2005). Rheumatic fever reappraised. *Chinese Medical Journal*.

[27] Pan ZG, Wang XN, Li YW, Zhang HY, Archard LC (2005). Detection of herpes simplex virus type 1 in rheumatic valvular tissue. *Chinese Medical Journal*.

[28] Soini Y, Salo T, Satta J (2003). Angiogenesis is involved in the pathogenesis of nonrheumatic aortic valve stenosis. *Human Pathology*.

[29] Isner JM, Chokshi SK, DeFranco A, Braimen J, Slovenkai GA (1990). Contrasting histoarchitecture of calcified leaflets from stenotic bicuspid versus stenotic tricuspid aortic valves. *Journal of the American College of Cardiology*.

[30] Vollebergh FEMG, Becker AE (1977). Minor congenital variations of cusp size in tricuspid aortic valves. Possible link with isolated aortic stenosis. *The British Heart Journal*.

[31] Hansson GK (2005). Mechanisms of disease: inflammation, atherosclerosis, and coronary artery disease. *The New England Journal of Medicine*.

[32] Mohler ER (2000). Are atherosclerotic processes involved in aortic-valve calcification?. *The Lancet*.

